# Intragenomic polymorphism and intergenomic recombination in the ribosomal RNA genes of strains belonging to a yeast species *Pichia membranifaciens*

**DOI:** 10.1080/21501203.2016.1204369

**Published:** 2016-07-11

**Authors:** Zuo-Wei Wu, Qi-Ming Wang, Xin-Zhan Liu, Feng-Yan Bai

**Affiliations:** State key Laboratory of Mycology, Institute of Microbiology, Chinese Academy of Sciences, Beijing, China

**Keywords:** Ribosomal RNA gene, intragenomic rDNA polymorphism, intergenomic rDNA recombination, concerted evolution, *Pichia membranifaciens*

## Abstract

A concerted evolution model has been proposed to explain the observed lack of sequence variation among the multiple ribosomal RNA (rRNA) gene copies in many different eukaryotic species. Recent studies on the level of intragenomic variations in the rRNA gene repeats of fungi resulted in controversial conclusions. In this study, we clearly showed that significant polymorphisms of the internal transcribed spacers (ITS1 and ITS2) of ribosomal DNA (rDNA) exist within the genome of a strain of the yeast species *Pichia membranifaciens*. More interestingly, we showed that the intragenomic ITS sequence polymorphisms were formed by intergenomic rDNA recombination among different *P. membranifaciens* strains with significantly different ITS sequences. Intergenomic rDNA recombination was also responsible for the diversification of rDNA sequences in different strains of the species. After the events bring together different rDNA types in individual genomes of the *P. membranifaciens* strains compared, rDNA sequence heterogeneity has remained in the genome of one but eliminated by homogenisation in the genomes of other strains. Our findings show new clue for further investigation on the mechanism of concerted evolution of rRNA genes in eukaryotes.

## Introduction

Ribosomal RNAs (rRNAs) are ubiquitous to all living organisms. Four rRNA genes, 26/28S, 18S, 5.8S and 5S, are present in eukaryotes. The 18S, 5.8S and 26/28S rRNA genes, separated by the two internal transcribed spacers (ITS1 and ITS2), are arranged in a transcription unit (Venema and Tollervey 1999). The rRNA unit is arrayed in tandemly repeated clusters with several hundred to a few thousand copies on one or separate chromosomes in the genome. It is generally believed that all the copies of the rRNA unit are identical or nearly identical in nucleotide sequence within the genome of a given organism because of the highly stringent functional constraints on rRNA molecules (Long and Dawid 1980; Hillis and Dixon 1991). A “concerted evolution” model has been proposed to explain the observed lack of sequence variation among rRNA gene copies in many different species (Zimmer et al. 1980; Coen et al. 1982; Liao 1999, 2000; Eickbush and Eickbush 2007).

However, exceptional cases have been reported in different organisms, including archaea, bacteria and eucaryotes (Mylvaganam and Dennis 1992; Wang et al. 1997; Yap et al. 1999). In eukaryotes, two types of 18S rRNA genes differing by 3.5% nucleotides were found within the individual genome of the parasite *Plasmodium berghei* (Gunderson et al. 1987). Two types of 18S rRNA genes differing by 8% nucleotides coexisted in the individual genome of the metazoan *Dugesia mediterranea* (Carranza et al. 1996). Intragenomic ribosomal DNA (rDNA) sequence polymorphisms have also been reported in fungi. Divergent ITS types have been found in single genomes of *Fusarium* and *Ganoderma* species (O’Donnell and Cigelnik 1997; Wang and Yao 2005). In a yeast species, *Clavispora lusitaniae*, intragenomic variations in the 26S rDNA D1/D2 domain have been reported (Lachance et al. 2003).

More recently, the level of intragenomic variations in the rRNA gene repeats of fungi were examined in two separate studies using whole-genome shotgun sequencing (WGSS) data, but the conclusions are inconsistent. Ganley and Kobayashi (2007) analysed the rDNA reads from WGSS projects of five fungi, including four ascomycetous species *Ashbya gossypii, Saccharomyces cerevisiae, Saccharomyces paradoxus* and *Aspergillus nudulans*, and one basidiomycetous species *Cryptococcus neoformans*. They found that the level of sequence variation within the rDNA arrays of these eukaryotic microorganisms was remarkably low, supporting the concerted evolution model. James et al. (2009) analysed the rDNA shotgun reads from the WGSS data sets for 34 strains of *S. cerevisiae*. Contrary to the result of Ganley and Kobayashi (2007), James et al. (2009) found that significant rDNA sequence variation exists within individual genomes and that the intragenomic variation can reach an order of magnitude between individual strains. Unexpectedly high intragenomic polymorphisms in nuclear ribosomal genes were also observed in four plant pathogenic ascomycetes species by Simon and Weiß (2008), who consequently assumed that nuclear ribosomal genes might not always evolve in a strictly concerted manner.

The origins of intragenomic rRNA gene polymorphisms in prokaryotes were explained by horizontal gene transfer (Yap et al. 1999). Gene conversion was shown to be the mechanism for homogenisation of rRNA genes in prokaryotes (Liao 2000). Compared to the tandem-arrayed organisation of eukaryotic rRNA genes in high copy number (generally over 100; in some cases >1000), prokaryotic rRNA genes are generally dispersed throughout a genome in limited copy numbers (generally <10) (Liao 2000). Since the concerted evolution theory has been proposed mainly based on sequence homogeneity in tandemly repeated multigene families (Eickbush and Eickbush 2007), the findings of intragenomic rRNA gene polymorphisms in eukaryotes are more challenging.

In an investigation on genetic diversity of strains belonging to a yeast species *Pichia membranifaciens*, remarkable intragenomic polymorphisms of the rDNA ITS regions were discovered. More interestingly, the intragenomic ITS sequence polymorphisms were clearly shown to be formed by intergenomic rDNA recombination among different *P. membranifaciens* strains. Intragenomic recombinations between the polymorphic ITS repeats were also observed. Furthermore, we found that intergenomic rDNA recombination is also responsible for the diversification of rDNA sequences in different strains of the species.

## Materials and methods

### Organisms, single-cell colony isolation and continuous cultivation

The yeast strains employed are listed in Table 1. They were obtained from the Centraalbureau voor Schimmelculture (CBS), The Netherlands. About 100 μl of adequately diluted actively growing cell suspension of a yeast strain was spread onto yeast extract-malt extract-peptone-glucose (Yarrow 1998) plate. The well isolated single cells were marked under a microscope. After incubation at 25°C for 48 h, the clearly isolated colonies developed from the marked single cells were picked for further study.

**Table 1.** Yeast strains studied.

Species/strain	Source	GenBank accession number for ITS
*Pichia membranifaciens*		
CBS 107	Exudation of elm	DQ104710
CBS 189	Unknown	DQ104712
CBS 191	Wine, Italy	DQ104713
CBS 212	Unknown	DQ104715
CBS 214	Grape must, Italy	DQ104716
CBS 215	Unknow	EF061127-EF061130
CBS 244	Banana	DQ104722
CBS 598	Lambic beer, Belgium	DQ104724
CBS 636	Sediment in lager tank	DQ104726
CBS 1329	Soil, Denmark	EF061131
CBS 8167	Gut of fly, Chile	EF061132
*Pichia mandshurica*		
CBS 209	Unknown	DQ104714
CBS 241	Unknown	DQ104718

*P. membranifaciens* strain CBS 215 which exhibits remarkable intragenomic polymorphisms in the rDNA ITS regions was subjected for continuous cultivation. The strain was grown in 200 ml of YPD (1% yeast extract, 2% peptone and 2% dextrose) medium and incubated at 25°C in a shaker (160 rpm). At the end of the exponential phase, 1 ml of the cell suspension was transferred to 200 ml new medium for continuing cell duplication. This procedure went on for 4 weeks until completion of around 400 generations. About 20 μl of cells taken at the end of approximately 100, 200, 300 and 400 generations were diluted and plated on YPD plates. Three to five well-isolated colonies were randomly picked for DNA sequence analysis.

### Genomic DNA extraction, PCR and direct sequencing

Genomic DNA of yeast strains were extracted by the method of Makimura et al. (1994). The DNA fragment covering the ITS1-5.8S-ITS2 region was amplified and sequenced with the primer pair ITS1 (5ʹ–GTC GTA ACA AGG TTT CCG TAG GTG–3ʹ) and ITS4 (5ʹ–TCC TCC GCT TAT TGA TAT GC–3ʹ).

### Cloning sequencing

Purified polymerase chain reaction (PCR) products of the ITS1-5.8S-ITS2 region from strain CBS 215 were cloned using the pUCm-T Vector system (BBI) and transformed into *E. coli* DH5α competent cells. Positive clones containing the target fragment were randomly picked for sequence analysis. The target fragments inserted in the plasmids were directly sequenced.

### Sequence alignment and phylogenetic analysis

Alignment of sequences was carried out with the Clustal X program (Thompson et al. 1997). The phylogenetic trees were constructed from the evolutionary distance data calculated from Kimura’s two-parameter model (Kimura 1980) by using the neighbour-joining method (Saitou and Nei 1987). Reference sequences were retrieved from GenBank under the accession numbers indicated in the phylogenetic tree.

### Pulsed-field gel electrophoresis

Intact yeast chromosomal DNA was prepared for pulsed-field gel electrophoresis (PFGE) by the method of Bai et al. (2000). Chromosomal DNA bands were separated in 0.8% (w/v) agarose gel in 0.5 × TBE (45 mM Tris/borate, 1 mM EDTA, pH 8.0) buffer in a contour-clamped homogeneous electric field (CHEF) electrophoresis apparatus (CHEF-Mapper XA, Bio-Rad, Hercules, CA, USA). Electrophoresis was performed for 51 h at 3 V/cm with pulse time ramping from 300 s to 600 s. The temperature of the running buffer was maintained at 12–14°C. After electrophoresis, the PFGE results were imaged with the AlphaImager 2200 gel documentation system (Alpha Innotech, San Leandro, CA, USA).

### Flow cytometry

Yeast cells for flow cytometric analysis were prepared using the method described in Burke et al. (2000) with minor modifications. Cells were grown in 3 ml of YPD broth at 28°C for 12 h and harvested from 300 μl of the culture for further processing. DNA was stained with propidium iodide and DNA content of yeast cells was determined using a BD FACSAria™ Flow Cytometer (Becton Dickinson, NJ, USA). *S. cerevisiae* strains FY1679-01B (haploid), FY1679-01D (haploid) and FY1679 (diploid) were used as reference strains.

## Results

### Intragenomic rDNA sequence polymorphisms in *P. membranifaciens* strain CBS 215

**Figure 1.**
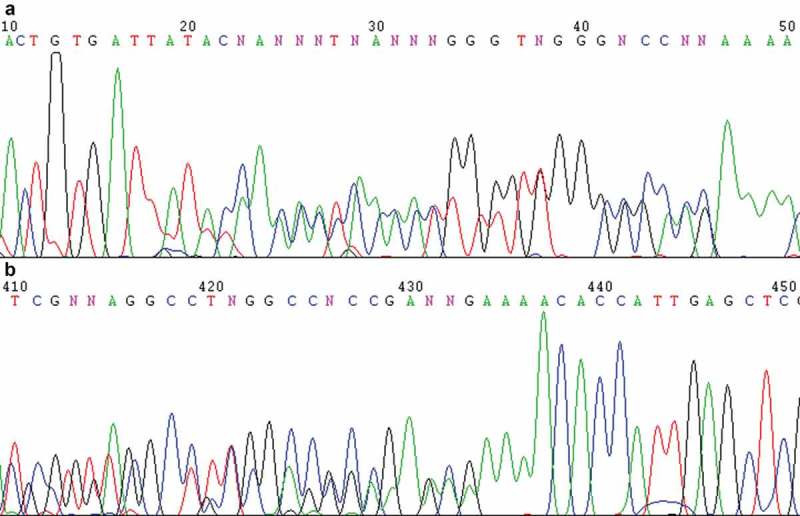
Direct sequencing chromatograms of the PCR product of the rDNA ITS1-5.8S-ITS2 region of *Pichia membranifaciens* strain CBS 215 using ITS1 (a) and ITS4 ((b), reverse complement of the original file) as the sequencing primers, showing the overlapped peaks.

The fragment covering the rDNA ITS1-5.8S-ITS2 regions of strain CBS 215 was amplified by PCR using nuclear DNA as the template. When we tried to determine the sequence of the purified PCR product by direct sequencing method, we found that overlapped peaks appeared after certain positions in the sequencing chromatograms using ITS1 or ITS4 as the sequencing primer (Figure 1). The phenomena indicated that non-homogenous sequences were included in the PCR products. Repeated purification of strain CBS 215 using the streak plate technique failed to eliminate the phenomena. In order to exclude the possibility of impurity, colonies developed from single cells were isolated sterilely by using single-cell colony isolation technique. Dozens of single-cell colonies were randomly selected for direct ITS region sequencing. The overlapped peaks appeared in each of the sequencing chromatograms of every colony at the same locations as in those of the original culture of strain CBS 215. The result indicated that the sequences of the rDNA ITS repeats within the genome of strain CBS 215 were polymorphic.

### Sequence variations among different ITS types within the genome of strain CBS 215

**Figure 2.**
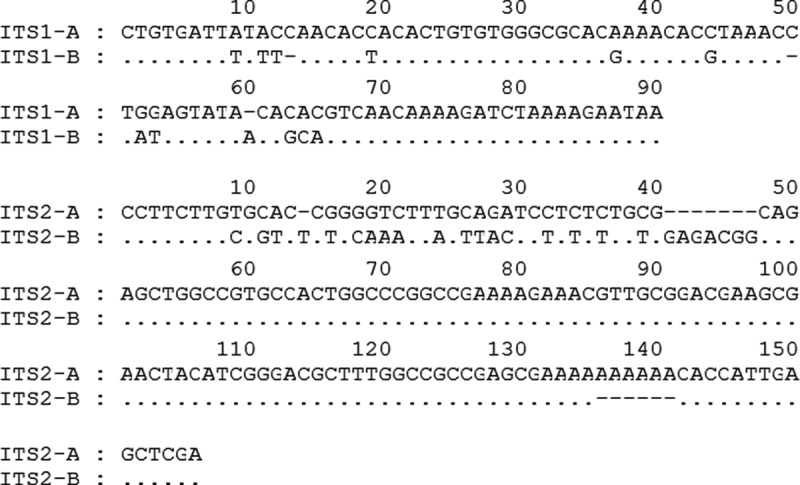
Sequence alignments of the different ITS types within the genome of *Pichia membranifaciens* strain CBS 215. At a given position, a nucleotide identical to that in the upper line is indicated by a dot, a gap is indicated by a hyphen.

The PCR product covering the ITS region (including the 5.8S gene) amplified from the DNA template from a single-cell colony of strain CBS 215 was cloned. A total of 107 clones containing target fragments were randomly selected and sequenced. Clear sequence chromatograms without overlapped peaks were obtained for every clone. Two types of ITS1 and two types of ITS2 were recognised from the clones (Figure 2). ITS1-A (89 bp in length, 76 clones) differed from ITS1-B (88 bp in length, 31 clones) by 14 mismatches (11 substitutions and 3 indels). ITS2-A (148 bp in length, 75 clones) differed from ITS2-B (150 bp in length, 32 clones) by 32 mismatches (18 substitutions and 14 indels) (Figure 2).

When the whole sequences of the ITS (ITS1-5.8S-ITS2) clones were compared, four types of ITS repeats formed by different combinations of the two types of ITS1 with the two types of ITS2 were recognised. Their sequences were type I (68 clones), ITS1-A + 5.8S + ITS2-A; type II (24 clones), ITS1-B + 5.8S + ITS2-B; type III (7 clones), ITS1-B + 5.8S + ITS2-A; and type IV (8 clones), ITS1-A + 5.8S + ITS2-B.

### Direct amplification of different ITS types from the genome of strain CBS 215

PCR amplification is know to be susceptible to the formation of chimeric products if the template DNA contains multiple divergent copies of the target fragment (Meyerhans et al. 1990; Wang and Wang 1996, 1997; Von Wintzingerode et al. 1997; Qiu et al. 2001; Boucher et al. 2004). Since the genome DNA from strain CBS 215 contained multiple divergent ITS repeats dominated by types I and II, the possibility that the clones of types III and IV were the chimera formed during PCR process could not be excluded, though the amplified fragment was less than 500 bp and chimera formation was less likely for such short products (Boucher et al. 2004).

In order to exclude this possibility, PCR primers were designed based on the variable sequences of different ITS1 and ITS2 types for specific amplification of the four types of ITS repeats from the genome DNA of strain CBS 215. Two forward primers ITS1-AF (5ʹ–CCG GCC ATC ATT ACT GTG ATT ATA CC–3ʹ) and ITS1-BF (5ʹ–GCG CGG ACT ATA GTA TAA CAG CA–3ʹ) and two reverse primers ITS2-AR (5ʹ–ATC TGC AAA GAC CCC GGT G–3ʹ) and ITS2-BR (5ʹ–AGC AGC TCT GCC GTC TCC AC–3ʹ) were designed. The primer pair ITS1-AF and ITS2-AR was used for specific amplification of type I; ITS1-BF and ITS2-BR for type II; ITS1-BF and ITS2-AR for type III; and ITS1-AF and ITS2-BR for type IV. PCR was performed at a very stringent condition to avoid non-specific amplification (36 cycles with denaturation at 94°C for 30 s, annealing at 69°C for 30 s and extension at 72°C for 20 s). The theoretic lengths of the fragments flanked by the primer pairs for ITS types I–IV are 287, 261, 233 and 315 bp, respectively.

**Figure 3.**
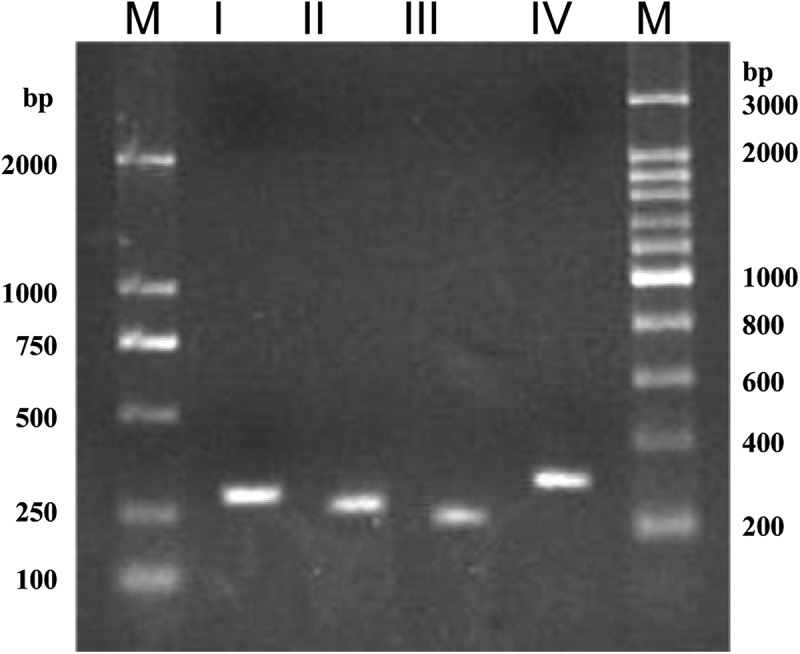
Specific amplification of rDNA ITS types I–IV from the genomic DNA of strain CBS 215. M, molecular size mark.

Clear amplicons with the sizes corresponding to the theoretic values were obtained from PCRs using each of the primer pairs and the DNA template of CBS 215 genome (Figure 3). Sequence analysis of each of the amplicons confirmed that the four types of ITS repeats were directly and specifically amplified. The result confirmed that in addition to the dominant ITS types I and II, types III and IV coexist in the genome of strain CBS 215.

### Sequence comparison of ITS repeats among different *P. membranifaciens* strains

**Figure 4.**
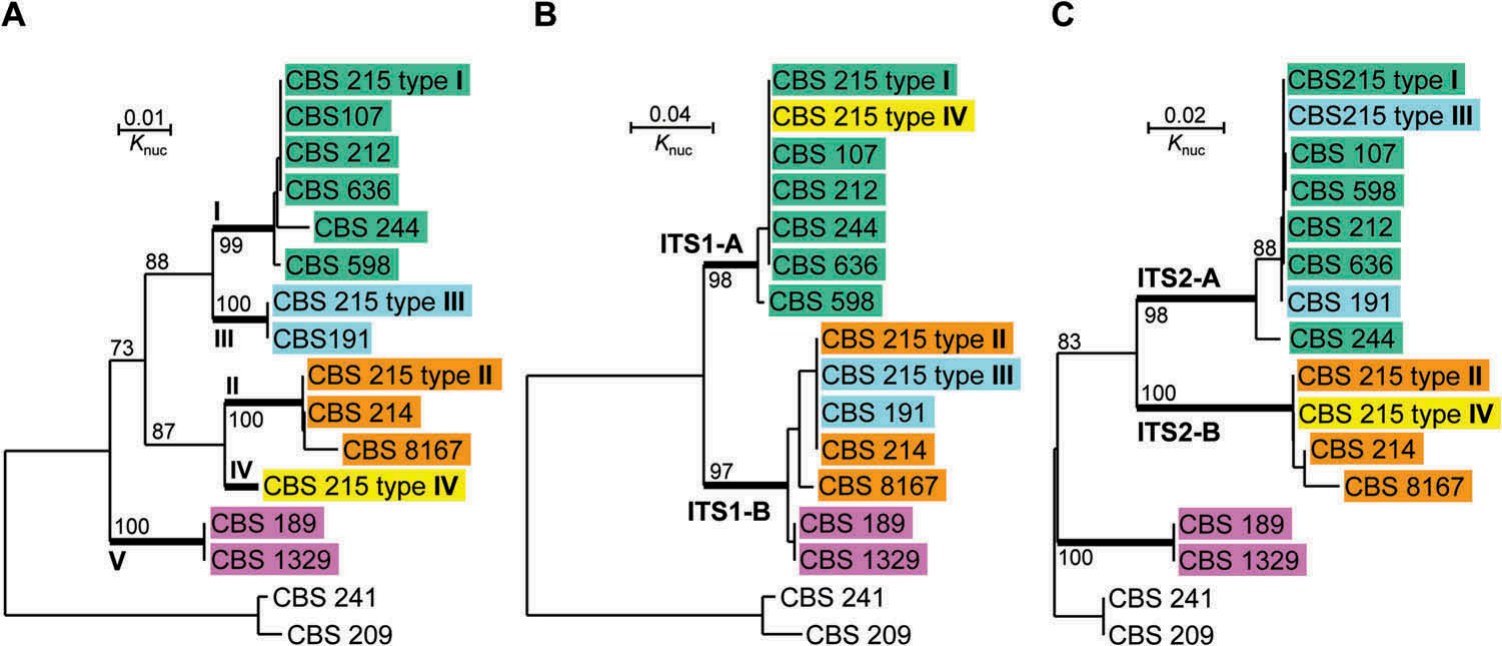
Neighbour-joining trees drawn from the sequence alignments of (a) the whole ITS repeat, (b) ITS1 only and (c) ITS2 only, of *Pichia membranifaciens* strains. *Pichia manchurica* strains CBS 209 and CBS 241 are used as the outgroup. Bootstrap percentages obtained from 1000 bootstrap replicates are shown.

In order to investigate the origin of the polymorphic ITS sequences in the genome of strain CBS 215, representatives of other strains of *P. membranifaciens* with different ITS sequences were selected for comparison. Intragenomic ITS polymorphisms were not found in these strains by direct sequencing (Wu et al. 2006). Evolutionary relationships of different ITS types of strain CBS 215 with those of other separate strains were depicted by phylogenetic trees drawn from the sequence alignments of the whole ITS fragment, ITS1 only and ITS2 only, respectively (Figure 4). Two strains of a closely related species *P. mandshurica* (Wu et al. 2006) were used as the outgroup. In the tree drawn from the whole ITS sequences, five clusters I–V were recognised. ITS types I–IV of CBS 215 were, respectively, distributed in the first four clusters. Cluster V was formed by strains CBS 189 and CBS 1329 (Figure 4(a)). In the ITS1 tree, two clusters corresponding to ITS1-A and ITS1-B sequences were recognised (Figure 4(b)). In the ITS2 tree, three clusters were formed (Figure 4(c)). The first two possessed ITS2-A and ITS2-B sequences, respectively. The third cluster consisted of strains CBS 189 and CBS 1329, indicating that they possess a unique ITS2 sequence, which is apparently responsible for their separation from the other strains in the tree drawn from the whole ITS sequences (Figure 4(a)). The base mismatches of different ITS1 and ITS2 types of strain CBS 215 with the corresponding ITS regions of related strains compared are summarised in Table 2.

**Table 2.** Sequence mismatches of different ITS1 and 2 types of strain CBS 215 with the corresponding ITS regions of closely related strains of *Pichia membranifaciens.*

	CBS 107	CBS 212	CBS 244	CBS 598	CBS 191	CBS 214	CBS 189
CBS 215							
ITS1-A (89 bp)	0	0	0	1	14	14	13
ITS1-B (88 bp)	14	14	14	13	0	0	2
ITS2-A (148 bp)	2	0	7	2	1	31	41
ITS2-B (150 bp)	31	32	25	31	30	1	22

The phylogenetic analysis and sequence comparison showed that each of the ITS1 and ITS2 types in strain CBS 215 exist separately in other *P. membranifaciens* strains (Figure 4(b) and 4(c); Table 2). In regard to the whole ITS repeat, types I, II and III in strain CBS 215 were also separately found from other independent strains (Figure 4(a)). Strains CBS 107, CBS 212 and CBS 244 possess ITS type I; strains CBS 214 and CBS 8167 possesses ITS type II and strain CBS 191 possesses ITS type III.

### Electrophoretic karyotyping and flow cytometry

**Figure 5.**
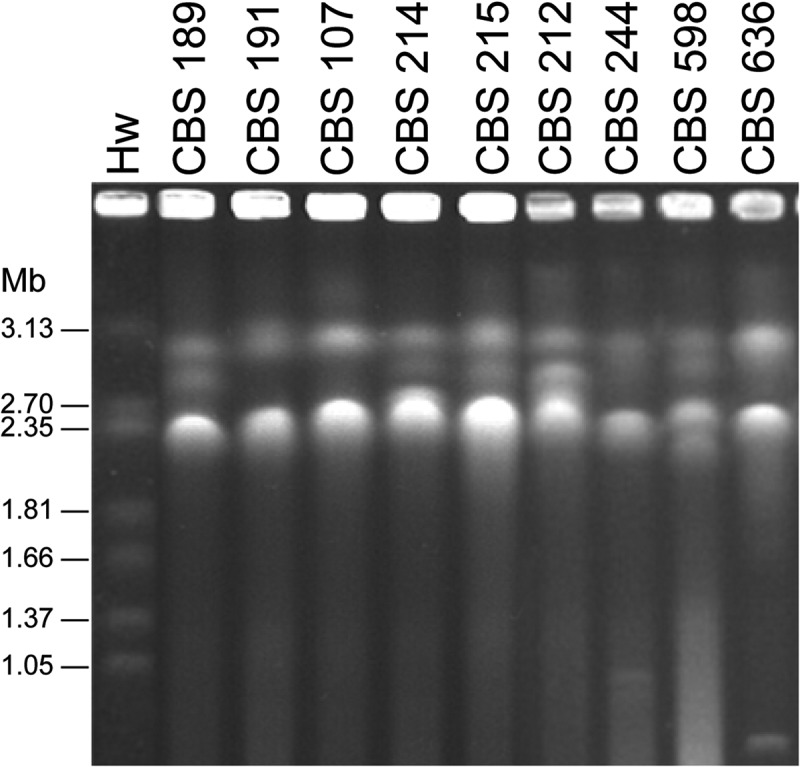
Electrophoretic karyotypes of *Pichia membranifaciens* strains. Hw, *Hansenula wingei* YB-4662-VIA.

Chromosomal DNA banding patterns of nine *P. membranifaciens* strains studied were compared (Figure 5). Two to four bands were resolved with molecular sizes ranging from 2.06 to 3.15 Mb. Strain CBS 244 appeared to have two additional small bands with molecular sizes of approximately 900 and 980 Kb, respectively. One additional small band (~780 Kb in length) was observed in strain CBS 636. Although the chromosome profiles of some of the strains compared were similar, none of them were identical. The result indicates that all of the strains compared represent differentiated strains, other than clones of single isolates.

**Figure 6.**
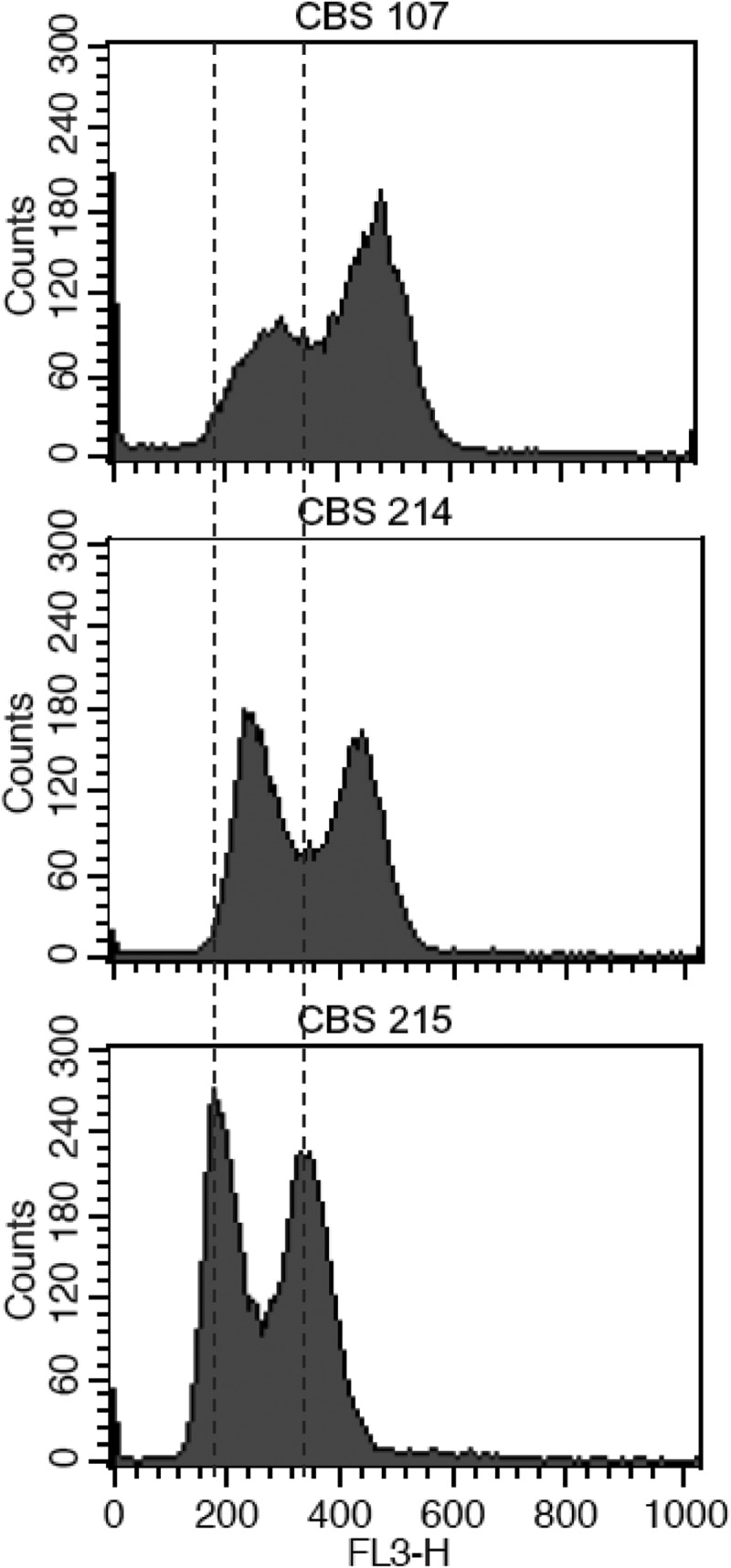
Flow cytometric analysis of *Pichia membranifaciens* strains CBS 107, CBS 214 and CBS 215.

As shown earlier, the polymorphic ITS types of strain CBS 215 come from different strains with different ITS sequences. It is possible that this strain is a diploid or polyploid organism simply formed by cell fusion of two or more strains with different ITS sequences. Flow cytometry analysis denied this assumption. Strains CBS 107 representing cluster I and CBS 214 representing cluster II were selected together with strain CBS 215 for comparative flow cytometry analysis. The level of fluorescence per cell as shown in Figure 6 indicates that the DNA content per cell of the latter is even slightly lower than those of the former two strains.

## Discussion

In this study, we clearly showed that significant polymorphisms of the rDNA ITS region exist within the genome of a strain (CBS 215) belonging to the yeast species *Pichia membranifaciens*. Four types of ITS repeat (ITS types I–IV) formed by different combinations of two types of ITS1 (ITS1-A and B) with two types of ITS2 (ITS2-A and B) were recognised in the genome of strain CBS 215. ITS1-A differs from ITS1-B and ITS2-A from ITS2-B by up to 15.6% and 20.5% base mismatches, respectively (Figures 2 and 3). Sequence comparison of different types of the whole ITS repeat, ITS1 and ITS2 of strain CBS 215 with the corresponding rDNA regions of other strains of the same species showed that (i) the polymorphic ITS sequences in the genome of strain CBS 215 originate from separate genomes of different strains and (ii) intergenomic recombination between different types of ITS repeats results in further diversification of the ITS sequences among different strains of *P. membranifaciens*.

The rDNA ITS region is one of the most frequently used molecular markers in phylogeny and identification of yeasts at the species level (James et al. 1996; Montrocher et al. 1998; Sugita et al. 1999a, b; Fell et al. 2000; Scorzetti et al. 2002; Kurtzman and Robnett 2003) and has been selected as a universal DNA barcode marker for *Fungi* (Schoch et al. 2012). Previous studies have claimed that conspecific yeast strains usually have fewer than 1% nucleotide differences in the ITS 1 and 2 regions overall (Nagahama et al. 1999; Sugita et al. 1999a, b; Scorzetti et al. 2002). However, the present study showed that divergent ITS1 and ITS2 types which differed by up to 15.6% and 20.5% base mismatches, respectively, coexist in the single genome of strain CBS 215. Each of the different ITS types distribute separately in other *P. membranifaciens* strains. Molecular data obtained in previous studies suggest that strains in the five clusters with remarkable divergent ITS sequences recognised in Figure 4(a) are apparently conspecific. The 26S rDNA D1/D2 sequences of the strains in these groups were identical or differed by only three or fewer substitutions (Wu et al. 2006). The DNA similarity values obtained from DNA/DNA hybridisation between strains CBS 107 (= IFO 10215) in cluster I, CBS 8167 (= IFO 10731) in cluster II and CBS 1329 (= IFO 10725) in cluster V, were 71–87% (Ueda-Nishimura and Mikata 2001). It is reasonable to expect similar or higher DNA similarity values between other strains from clusters I to V. Comparison of genetic cross and DNA reassociation has indicated that yeast strains showing DNA similarity at 70% or higher are usually conspecific (Kurtzman 1998). Our data suggest that conspesific yeast strains may occasionally have significantly divergent ITS sequences.

Sequence comparison of multiple independent strains clearly indicates that the polymorphic ITS sequences of strain CBS 215 arise from different strains with different ITS types. Since the majority of the strains compared possess ITS type I only (cluster I in Figure 4(a)) and the dominant ITS types in strain CBS 215 are types I and II, the most possibility is that ITS type I of strain CBS 215 is from a strain in cluster I and type II from a strain in cluster II. The possibility that the other two minor ITS types III and IV in the genome of strain CBS 215 also come from different stains (e.g. type III from a strain in cluster III and type IV from a strain in Cluster IV) cannot be excluded. However, this possibility may be very low, because the process needs multiple hybridisation or similar events and strains with ITS type III or IV only may be rarely distributed in nature (one strain with ITS type III only but none with ITS type IV only has been recognised). Therefore, we think that the minor ITS types III and IV in the genome of strain CBS 215 are most possibly formed by intragenomic recombination between the two dominant types I and II.

The independent distribution of each of ITS types I to III in separate strains is clearly shown in this study (Figure 4(a)). It is reasonable to infer that ITS type IV also distributes independently in other strains in nature. Since the four ITS types (I–IV) are apparently formed by different combinations of two types of ITS1 with two types of ITS2 (Figure 4), the reasonable explanation to the independent occurrence of each of the four ITS types in separate strains is intergenomic recombination. For instance, the ITS repeat of strain CBS 191 which possesses type III only may be formed by intergenomic recombination between two strains with ITS types I and II, respectively. The sequence similarity of ITS1 of strains CBS 189 and CBS 1329 to ITS1-B suggests that the ITS repeat of these two strains may be formed by intergenomic recombination between the ITS repeats from a strain with ITS1-B and a strain with another unique ITS2 type (Figure 4).

Intragenomic polymorphisms of the ITS region have also been reported in other eukaryotes, including plants (Baldwin et al. 1995; Alvarez and Wendel 2003), beetles (Vogler and DeSalle 1994), nematodes (Zijlstra et al. 1995), sponges (Wörheide et al. 2004) and filamentous fungi (O’Donnell and Cigelnik 1997; Wang and Yao 2005). The divergent ITS types in the same individual genomes of these organisms are usually described as nonorthologous and attributed to the origin of ancient interspecific hybridisation (xenologous origin) or gene duplication (paralogous origin), but direct evidences have not been presented. Recombination or gene conversion in the ITS region has been observed in a hybrid of a mushroom (Hughes and Petersen 2001). The hybrid was recognised as being formed by a natural hybridisation event between two separate species of *Flammulina* (*F. velutipes* and *F. rossica*) which resulted in a homogenised ribosomal repeat containing elements of both parents (Hughes and Petersen 2001). The present study clearly showed that the ITS heterogeneity of strain CBS 215 arises from separate strains with different ITS sequences in the same species. An event of mating, fusion or hybridisation between strains with different ITS types may account for the phenomenon. Flow cytometry analysis showed that strain CBS 215 is not simply a diploid or polyploid yeast formed by fusion of two or more cells of two or more different strains (Figure 6). Therefore, the exact way that brings together different ITS types into a single genome is uncertain.

As discussed earlier, the ITS repeats of strains CBS 198, CBS 191 and CBS 1329 are apparently formed by intergenomic recombination of different ITS types from different strains. However, intragenomic ITS sequence polymorphisms have not been detected in any of these strains. It is interesting to note that, after the events bringing together different rRNA gene repeats into single genomes, concerted evolution has occurred in strains CBS 189, CBS 191 and CBS 1329, but not in strain CBS 215. The genome of strain CBS 215 seems stable, since ITS sequence polymorphisms maintained in repeated subcultures and in all randomly selected colonies of the strain after repeated purification using the plate streak method. The escape of rRNA gene repeats of strain CBS 215 from homogenisation by concerted evolution is challenging. One possible explanation is the defect in gene conversion mechanisms required for concerted evolution of rRNA genes in strain CBS 215. The hypotheses proposed to explain long-term persistence of rDNA polymorphsims following hybridisation or polyplodisation have been summarised by O’Donnell and Cigelnik (1997) as (1) their chromosomal location may not be telomeric, (2) the sequences may have diverged beyond the point that interchromosomal conversion can occur and (3) concerted evolution may be more efficient within a chromosomal locus than between repeats dispersed on non-homologous chromosomes. Rooney (2004) has shown that the intragenomic heterogeneous 18S rRNA genes in apicomplexan protists undergo birth-and-death rather than concerted evolution. In these protists, rRNA genes are dispersed throughout the genome and are not organised in a tandem array. More recently, Rooney and Ward (2005) revealed that the dispersed 5S rRNA genes of filamentous fungi also evolve under a birth-and-death model with strong purifying selection and rapid gene turnover. Strain CBS 215 with intragenomic heterogeneous ITS repeats and strains CBS 189, CBS 191 and CBS 1329 with homogenous ITS repeats are closely related within a single yeast species. It will be interesting to compare rRNA gene organisation and structure of these strains using high-quality genome sequencing. The results will certainly be helpful to elucidate the mechanism allowing the maintenance of the rDNA polymorphsims in strain CBS 215, and in return, to have a better understanding of the concerted evolution model of rRNA genes.
